# Performance Analysis of ICA in Sensor Array

**DOI:** 10.3390/s16050637

**Published:** 2016-05-05

**Authors:** Xin Cai, Xiang Wang, Zhitao Huang, Fenghua Wang

**Affiliations:** College of Electronic Science and Engineering, National University of Defense Technology (NUDT), Changsha 410073, China; christopherwx@163.com (X.W.); huangzhitao@nudt.edu.cn (Z.H.); wfh.abc@163.com (F.W.)

**Keywords:** blind source separation, independent component analysis, performance analysis, practical factors

## Abstract

As the best-known scheme in the field of Blind Source Separation (BSS), Independent Component Analysis (ICA) has been intensively used in various domains, including biomedical and acoustics applications, cooperative or non-cooperative communication, *etc.* While sensor arrays are involved in most of the applications, the influence on the performance of ICA of practical factors therein has not been sufficiently investigated yet. In this manuscript, the issue is researched by taking the typical antenna array as an illustrative example. Factors taken into consideration include the environment noise level, the properties of the array and that of the radiators. We analyze the analytic relationship between the noise variance, the source variance, the condition number of the mixing matrix and the optimal signal to interference-plus-noise ratio, as well as the relationship between the singularity of the mixing matrix and practical factors concerned. The situations where the mixing process turns (nearly) singular have been paid special attention to, since such circumstances are critical in applications. Results and conclusions obtained should be instructive when applying ICA algorithms on mixtures from sensor arrays. Moreover, an effective countermeasure against the cases of singular mixtures has been proposed, on the basis of previous analysis. Experiments validating the theoretical conclusions as well as the effectiveness of the proposed scheme have been included.

## 1. Introduction

The fundamental goal of BSS is to recover the original signals from their mixtures when the mixing process is unknown. Since first considered in the biological problem of motion decoding in vertebrates by Jutten, Ans and Roll [1], the BSS problem has been expanded in many multi-sensor systems: Antenna arrays in electromagnetism or acoustics, chemical sensor arrays, electrode arrays in electroencephalography, *etc.* This very wide set of possible applications is probably one reason for its success [1,2]. Independent Component Analysis (ICA) is one of the most widely used BSS techniques for revealing hidden factors that underlie sets of random variables, measurements, or signals. The ICA of a random vector consists of searching for a linear transformation that minimizes the statistical dependence between its components [3,4]. The power of ICA resides in the physical assumptions that different physical processes generate unrelated signals. The simple and generic nature of this assumption allows ICA to be successfully applied in a diverse range of research fields [5]. For the last three decades, ICA has received attention from varied domains, including the biomedical applications [6,7,8,9,10,11,12,13] such as the single-channel electromyogram (EMG) classification with ensemble-empirical-mode-decomposition-based ICA for diagnosing neuromuscular disorders in [6], the identification of simple and complex finger flexion movements using surface electromyography (sEMG) and muscle activation strategy with Subband Decomposition ICA (SDICA) in [7] and the driver fatigue classification with ICA by Entropy Rate Bound Minimization (ICA-ERBM) in an electroencephalography (EEG)-based system in [8]; the audio source separation like the noisy speech recognition [14], the multichannel blind deconvolution and speech separation [15]; the cooperative or non-cooperative communication like the semi-blind signal extraction with possible constrains [16], the blind adaptive detection in systems like Direct Sequence Code Division Multiple Access (DS-CDMA) [17]; other applications also include those in chemical sensor arrays [18], the target decomposition (TD) in image processing [19] and the estimation of modal parameters in vibration systems [20].

There has been numerous algorithms that belong to the ICA scheme. Most representative ones are: (1) the FastICA algorithm by Hyvärinen and Oja [5,21,22], which is a fixed point algorithm that employs higher order statistics for the recovery of independent sources. FastICA uses simple estimates of negentropy based on the maximum entropy principle, which requires the use of appropriate nonlinearities for the learning rule of the neural network; (2) the Equivariant Adaptive Separation via Independence (EASI) algorithm by Cardoso and Laheld [23] that is a class of adaptive algorithms for source separation implementing an adaptive version of equivariant estimation. The EASI algorithms are based on the idea of serial updating; (3) the Infomax algorithm by Bell and Sejnowski [24], which belongs to a self-organizing learning algorithm maximizing the information transferred in a network of non-linear units, and is also regarded as a higher-order generalization of the principal component analysis (PCA); (4) The Joint Approximate Diagonalization of Eigenmatrices (JADE) algorithm by Cardoso and Souloumiac [25,26] consisting of high-order measures of independence and the class of Jacobi algorithms for their optimization. In the JADE algorithm, joint diagonalization of cumulant matrices allows the whole high-order cumulant set to be processed with computational efficiency similar to eigen-based techniques.

Because of its extensive applications and attractive properties, the performance of ICA algorithms has also been studied by many researchers. Some early works mainly centered on the fundamental problem of separability, meaning whether there is the possibility for the mixtures to be separated. Several criteria have been put forth based on the matrix theory or the structure characterization theory of random variables [27,28,29]. For instance, the concept of the *m*-row decomposability is introduced in [27] and it is proved therein that if the mixing matrix is *m*-row decomposable, then in principle it is possible to separate the source signals into *m* groups. In [28], the authors proved that the signals can be decoupled, or separated, using only the condition that they are statistically independent, and found even weaker sufficient conditions involving their cross-polyspectra. The results of these works are significant, but they lack further discussion on the separation quality when decoupling is possible, which is crucial and of great concern for the applications. The work had been carried on by several researchers. In [30], Cardoso studied the most common class of orthogonal invariant algorithms for the blind separation of independent sources in terms of performance, quantified by the rejection rate, and a pairwise lower bound on the performance was given. Comon analyzed the theoretical asymptotic performance of a contrast-based BSS scheme in [31]. Finally, in [32], the Cramér-Rao lower bounds for linear ICA as an algorithm independent theoretical limit of the achievable separation quality was deduced. Limitations and performance bounds of certain ICA algorithms have been indicated in these works, but little attention has been paid to the actual performance under different situations depicted by the mixing processes, the sources as well as the environment, while the issue is actually significant for the applications. Analysis of the performance of several ICA algorithms under varied conditions was referred to in the scenario of blind suppression of interfering signals in direct sequence spread spectrum communication systems [33], but the work was mainly based on computer simulations with little in the way of analytical expression and conclusions.

Generally speaking, we think it is of great significance to analyze the performance of ICA algorithms under varied conditions depicted by the mixing matrix, the sources as well as the environment, since conclusions (especially quantitative ones) from such analysis may act as guidance for the applications of wide variety. For instance, it should be helpful to learn how to design the receiving array so as to expect better separation performance and more robustness against intricate situations? Why the algorithm works well for some mixtures while being unsatisfactory for the others? Under what kind of scenarios would the ICA algorithms fail? and further, what measures can be taken when faced with such failure? Answers to such questions are interesting and of great importance. However, little attention has been paid to the issue in the current literature, especially analytical form works.

In this manuscript, the typical scenario of antenna array receiving is taken as an illustration for the varied applications involving sensor arrays. The problem of concern is the performance of ICA algorithms under varied situations depicted by the mixing matrix, the sources and the environment noise. The influence of several fundamental factors in practice has been analyzed with quantitative results; factors concerned include the environment noise level, the element spacing of the array, the frequencies of the sources as well as their locations. The analytic connection between the noise variance, the source variance, the condition number of the mixing matrix and the optimal signal to interference-plus-noise ratio is given. Also, the question how the mixing matrix is affected by the element spacing of the array, the frequencies and locations of the radiators is considered, with special concern on the prevention of (nearly) singular mixing matrices. What is more, an effective countermeasure against the situations of singular mixtures has been proposed, on the basis of previous analysis. The manuscript is arranged as follows. Models of the noisy ICA and the sensor array output as well as the definition of the condition number are introduced in Section 2. Analytical works are mainly implemented in Section 3. In Section 4, simulation works are carried out to demonstrate the validity of previous results and conclusions. Summaries and conclusions are stated in Section 5.

## 2. Hypotheses, Definitions and Notations

### 2.1. Noisy ICA Model

Denote x a data matrix. We think of it as a *P* × *L* object collecting *L* samples of a *P* × 1 vector, the model of independent component analysis postulates a number *N* of sources so that the observation matrix x can be explained as:

(1) x=As+V

where **A** is the unknown *P* × *N* mixing matrix, s is a *N* × *L* matrix in which each row is called a ’’source signal” or a “component”. Matrix **V** represents an additive noise item or some other form of measurement uncertainty [1]. ICA is distinguished from other approaches to source separation in that it requires relatively few assumptions on the sources and on the mixing process. Basic assumptions of ICA include [5]:

(1) The sources being considered are statistically independent.

The first assumption is fundamental to ICA. Statistical independence is the key feature that enables estimation of the independent components from the observations.

(2) The independent components have non-Gaussian distribution.

The second assumption is necessary because of the close link between Gaussianity and independence. It is impossible to separate Gaussian sources using the ICA framework because the sum of Gaussian sources is indistinguishable from a single Gaussian source, and for this reason Gaussian sources are forbidden. This is not an overly restrictive assumption as in practice most sources of interest are non-Gaussian.

(3) The mixing matrix is invertible.

The third assumption is straightforward. If the mixing matrix is not invertible then clearly the demixing matrix we seek to estimate does not even exist.

Generally speaking, the ICA scheme aims at estimating a demixing matrix **B** based on the output independence that satisfies [3,4,5]:

(2) $$B=PDA^{-1}$$

where **P** is a permutation matrix, **D** is a diagonal matrix, and the superscript ‘(□)^–1^’ indicates the matrix inversion. The estimation of sources (with intrinsic scale-permutation ambiguity) is obtained with:

(3) $$\hat{s}=Bx$$

### 2.2. Sensor Array Output

**Figure 1:**
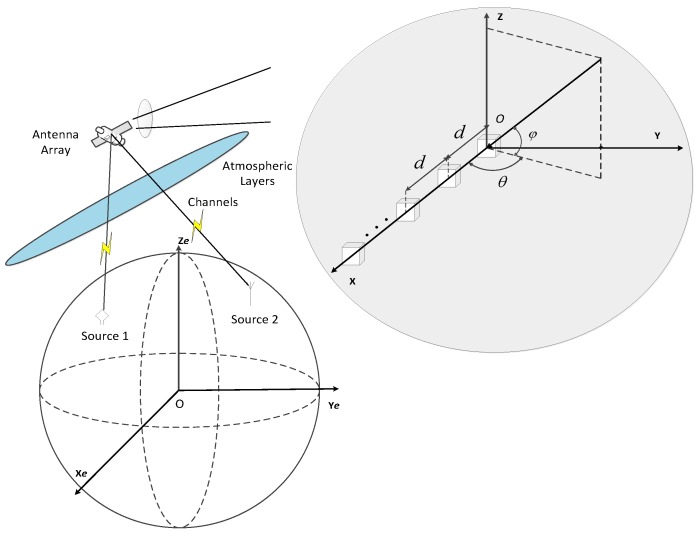
Spaceborne non-cooperative communication with antenna array.

In this manuscript, the typical scenario of a receiving antenna array such as in spaceborne non-cooperative communication is taken as an illustration for the varied applications involving sensor arrays, as depicted by Figure 1.

The array consists of *P* narrowband (NB) omnidirectional antennas and we call **x**(*t*) the vector of complex amplitude of signals at the output. Each antenna is assumed to receive the contribution of *N* zero-mean stationary NB sources that are statistically independent. Under these assumptions, we model **x**(*t*) as:

(4) $$x(t)=\sum_{i=1}^{N}\alpha_{i}(t)\cdot e^{j2\pi v_{i}t}\cdot e^{-j2\pi f_{i}\tau_{i}(t)}\cdot s_{i}(t)\cdot a_{i}(t)+v(t),\;t\in [0,T]$$

where “*j*” is the imaginary unit, *T* is the observation length, *α_i_*(*t*), *υ_i_* and *τ_i_*(*t*) are respectively the attenuation, the frequency offset and the propagation delay of the channel corresponding to source *i* at time *t*; *f_i_* is the carrier frequency of the NB source *i*; *s_i_*(*t*) is the complex amplitude of the corresponding source; **a***_i_*(*t*) is the equivalent steering vector (or spatial signature) of source *i* at time *t*, which is a *P* × 1 vector depending on the array, the direction of arrival (DOA) and is also relevant with carrier frequencies of sources. **v**(*t*) is the noise vector, assumed to be zero-mean, stationary, circular, Gaussian and spatially white, with variance $\sigma_{v}^{2}$ in the sampling band of frequency. In addition, the array is set as a uniform linear array (ULA), the element spacing of which is *d*, and sensors are disposed along the (*Ox*) axis. Notice that the sources are presumed to be statistically independent, which is generally the case for physically separated sources in reality.

Regarding the first unit of the array as the datum unit and denoting *N* complex equivalent sources arriving at the datum unit as ${\tilde{s}}_{i}(t)$, we have:

(5) $${\tilde{s}}_{i}(t)≜\alpha_{i}(t)\cdot e^{j2\pi v_{i}t}\cdot e^{-j2\pi f_{i}\tau_{i}(t)}\cdot s_{i}(t)$$

Then Equation (4) can be rearranged as:

(6) $$x(t)=\sum_{i=1}^{N}{\tilde{s}}_{i}(t)\cdot a_{i}(t)+v(t),\;t\in [0,T]$$

Regarding the steering vector to be constant within the observation window, then **a***_i_*(*t*) can be explicitly expressed as:

(7) $$\begin{array}{l} a_{i}(t)=a_{i}(\theta_{i},\varphi_{i},\lambda_{i})={\left[1,a_{i2}(\theta_{i},\varphi_{i},\lambda_{i}),...,a_{iP}(\theta_{i},\varphi_{i},\lambda_{i})\right]}^{T}\\ =\exp\left\{\frac{j2\pi \left[x\cdot cos(\theta_{i})\cdot cos(\varphi_{i})+y\cdot sin(\theta_{i})\cdot cos(\varphi_{i})+z\cdot sin(\varphi_{i})\right]}{\lambda_{i}}\right\} \end{array}$$

where *θ_i_* and *ϕ_i_* are the azimuth and elevation incident angles of the source *i*, *θ_i_* ∈[−90°, 90°]; $\varphi_{i}\in [-90^{o},\;90^{o}]$; *λ_i_* is the wavelength corresponding to the carrier frequency of the NB source *i*, *λ_i_* = *c*/*f_i_* (*c* denotes the speed of light); (**x**,**y**,**z**) are the coordinates of sensors in the array, as depicted in Figure 1; the superscript “*T*” denotes transpose.

**Figure 2:**
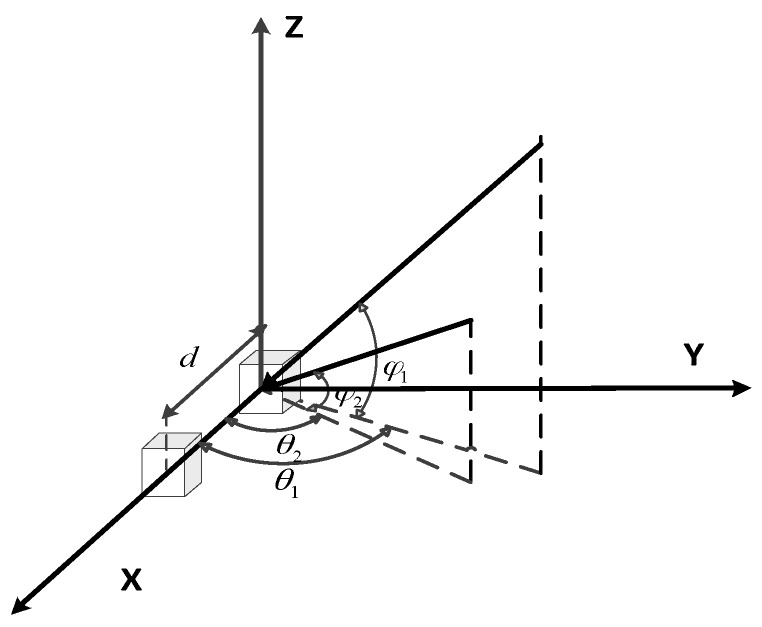
Illustration of the case *P* = *N* = 2.

Without the loss of generality, we consider the fundamental situation where *P* = *N* = 2. The coordinate of the second sensor is (*d*,0,0) and DOAs of sources are (*θ*_1_,*ϕ*_1_) and (*θ*_2_,*ϕ*_2_) respectively, as depicted by Figure 2. Moreover, the frequency offsets of sources are supposed to be negligible, and the propagation channels are assumed to be time invariant over the observation duration. Hence, the mixing matrix **A** whose columns are vectors **a***_i_*(*θ_i_*,*ϕ_i_*_,_*λ_i_*) will be:

(8) $$A=\left[\begin{array}{cc} 1 & 1\\ \exp\left\{\frac{j2\pi d\cdot cos\theta_{1}\cdot cos\varphi_{1}}{\lambda_{1}}\right\} & \exp\left\{\frac{j2\pi d\cdot cos\theta_{2}\cdot cos\varphi_{2}}{\lambda_{2}}\right\} \end{array}\right]$$

### 2.3. Condition Number of Matrix

Consider a system of linear equations:

(9) x=As

The system is featured by the coefficient matrix **A**, **x** is the vector of data, and **s** is the solution vector to be estimated.

*Definition 1* [34]:

The condition number of a non-singular matrix **A**, denoted as *cond*(**A**), can be calculated via:

(10) $$cond(A)≜\left\|A\right\|\left\|A^{-1}\right\|$$

where ′‖•‖′ indicates certain kind of matrix norm. For singular matrix **G**, *cond*(**G**) = ∞.

The condition number measures the singularity of a matrix or equivalently, the sensitivity of the solution of corresponding linear equation system to errors in the data. Hence, it is a valid indicator of the accuracy and numerical stability of results from matrix inversion and linear equation solution. When a minor perturbation in the observation only leads to a small error in the solution vector, the matrix **A** is considered to be well-conditioned, and inversely, if minor perturbation of **x** results in large error in the estimation of **s**, then the matrix is said to be ill-conditioned.

The connotation of the condition number can be understood in a more straightforward way, that is, it measures the amplification between errors in **x** (denoted as ∆**x**) and that in the estimation of **s** (denoted as ∆**s**). In fact, it has been proved in [34] that:

(11) $$\frac{\left\|∆s\right\|}{\left\|s\right\|}\leq cond(A)\frac{\left\|∆x\right\|}{\left\|x\right\|}$$

In this manuscript, we adopt the Frobenius norm for the calculation of the condition number, which is defined as [34]:

(12) $${\left\|A_{P\times N}\right\|}_{F}≜{\left(\sum_{i=1}^{P}\sum_{k=1}^{N}{\left|a_{ik}\right|}^{2}\right)}^{\frac{1}{2}}$$

in which *a_ik_* is the element of **A** in the *i*-th row and *k*-th, ′|·|′ indicates the module value.

Combining Equations (8), (10) and (12), the condition number in the 2 × 2 array receiving case is obtained as:

(13) $$cond(A)=\frac{4}{\sqrt{2-2cos[2\pi d\cdot (\frac{cos\theta_{2}cos\varphi_{2}}{\lambda_{2}}-\frac{cos\theta_{1}cos\varphi_{1}}{\lambda_{1}})]}}$$

## 3. Performance of ICA *vs.* Practical Factors

### 3.1. Environment Noise and the Condition Number

Since restitution of sources via typical ICA algorithms equals solving the linear equation set in nature, it has been well-known that the performance of ICA estimators in the noisy case depends on the condition number of the mixing matrix and the environment noise level. More specifically, it should be intuitively understood that the ICA algorithms would be more sensitive to the noise item when the singularity of the mixing matrix is stronger. Here, we attempt to push the work further via figuring out the upper bound of the ICA separation performance, under certain noise level and the condition number.

The concept of the optimal separator that BSS methods aim at implementing asymptotically is introduced in [35], which indicates the upper limit of the separation performance for all ICA algorithms. In [35], the separation performance is measured by the criterion signal to interference-plus-noise ratio (SINR), and the SINR of source *k* at output *i* of separator **B** is defined therein as:

(14) $${\text{SINR}}_{k}(b_{i})≜\sigma_{{\tilde{s}}_{k}}^{2}\frac{{\left|b_{i}^{}a_{k}\right|}^{2}}{b_{i}R_{\backslash k}b_{i}^{H}}$$

where **b***_i_* is the *i*-th row of **B**, $\sigma_{{\tilde{s}}_{k}}^{2}=E\{{\left|{\tilde{s}}_{k}(t)\right|}^{2}\}$ (′E{·}′ stands for mathematical expectation), ${\tilde{s}}_{k}(t)$ is the *k*-th equivalent source in Equation (5), **a***_k_* is the steering vector of source *k*, **R***_k_* is the total noise correlation matrix temporal mean for the source *k*, defined by $R_{\backslash k}≜R_{x}-\sigma_{{\tilde{s}}_{k}}^{2}a_{k}a_{k}^{H}$, $R_{x}≜E\{x(t)x{\left(t\right)}^{H}\}$ is the auto-correlation of the observation, and the superscript “*H*” indicates conjugate transpose.

The restitution quality of the source *k* by the separator **B** can be evaluated by the quantity SINRM*_k_*, which is the maximum value of SINR*_k_*(**b***_i_*) among all 1 ≤ *i* ≤ *N*. It has been verified in [35] that under this criterion, **B** and **BΛΠ** have the same performance, in which **Λ** is a diagonal matrix and **Π** is a permutation matrix. This indicates that the criterion itself is not susceptible to the intrinsic scale-permutation ambiguity of the ICA.

For the case of two sources, the SINRM_1_ at the output of the optimal separator is given by:

(15) $${\text{SINRM}}_{1}=\varepsilon_{1}(1-\frac{\varepsilon_{2}}{1+\varepsilon_{2}}{\left|\alpha_{12}\right|}^{2})$$

where $\varepsilon_{k}≜\sigma_{{\tilde{s}}_{k}}^{2}a_{k}^{H}R_{v}^{-1}a_{k}$ denotes the signal-to-noise ratio (SNR) at the input of the separator for source *k*, **Rv** is the auto-correlation of the noise item. *α*_12_ is the spatial correlation coefficient between sources 1 and 2 in the matrix of $R_{v}^{-1}$, defined by:

(16) $$\alpha_{12}≜\frac{a_{1}^{H}R_{v}^{-1}a_{2}}{{\left(a_{1}^{H}R_{v}^{-1}a_{1}\right)}^{1/2}{\left(a_{2}^{H}R_{v}^{-1}a_{2}\right)}^{1/2}}$$

One thing to be noticed is that when the noise on the sensors are additive white Gaussian (as presumed in this paper), we have $R_{v}=\sigma_{v}^{2}I$ (where **I** is the unit matrix) and thus $\varepsilon_{k}=\left(\sigma_{{\tilde{s}}_{k}}^{2}/\sigma_{v}^{2}\right)a_{k}^{H}a_{k}$. With Equation (8), the expression can be further simplified as:

(17) $$\varepsilon_{k}=\left(2\sigma_{{\tilde{s}}_{k}}^{2}/\sigma_{v}^{2}\right)$$

and Equation (16) may be transformed as:

(18) $$\alpha_{12}=\frac{a_{1}^{H}a_{2}}{{\left(a_{1}^{H}a_{1}\right)}^{1/2}{\left(a_{2}^{H}a_{2}\right)}^{1/2}}$$

Combining Equations (8) and (18), we have:

(19) $$\left|\alpha_{12}\right|=\frac{1}{2}\sqrt{2+2cos[2\pi d\cdot (\frac{cos\theta_{2}\cdot cos\varphi_{2}}{\lambda_{2}}-\frac{cos\theta_{1}\cdot cos\varphi_{1}}{\lambda_{1}})]}$$

With Equations (13), (15) and (19), there is:

(20) $${\text{SINRM}}_{1}=\varepsilon_{1}\left[\frac{1}{1+\varepsilon_{2}}+\frac{4\varepsilon_{2}}{1+\varepsilon_{2}}\cdot \frac{1}{cond^{2}(A)}\right]$$

and when source 2 is strong enough ($\varepsilon_{2}\gg \;1$), Equation (20) can be simplified with approximation as:

(21) $${\text{SINRM}}_{1}\approx \frac{4\varepsilon_{1}}{cond^{2}(A)}$$

Similar expression can be obtained for SINRM_2_.

Equations (20) and (21) indicate that the superior limit of the separation performance via ICA algorithms is mainly determined by the SNR at the input of the separator and the condition number of the mixing matrix. Generally speaking, the performance of the algorithms would be more susceptible to the noise with larger condition number of the mixing matrix.

According to Equation (13), *cond*(**A**) ≥ 2, and the condition number reaches its minimum, which indicates the weakest singularity of the mixing process, it is straightforward that $cos[2\pi d\cdot (\frac{cos\theta_{2}\cdot cos\varphi_{2}}{\lambda_{2}}-\frac{cos\theta_{1}\cdot cos\varphi_{1}}{\lambda_{1}})]=-1$ and $\left|\alpha_{12}\right|=0$. Then according to Equation (15), the SINRM_1_ reaches its maximum and equals *ε*_1_ regardless of *ε*_2_, which indicates that the SINR at the output equals the SNR at the input of the separator. Since this is theoretically the best result possible, *cond*(**A**) = 2 should be the ideal case that is desired in practice.

On the contrary, the case that should be avoided is the singular mixtures, meaning the situation where *cond*(**A**) = ∞. This is because then the multi-channel ICA problem would degenerate into a single-channel one, which the traditional ICA algorithms cannot handle, and thus, the separation may come to a critical failure in applications.

### 3.2. Condition Number vs. Practical Factors

It can be seen from Equation (17) that under the white Gaussian noise assumption, the SNR at the input of the separator is only determined by the variances of the sources and the noise. That is to say, factors including the element spacing of the array, the frequencies and locations of the NB sources would not affect the signal-to-noise ratio, thus these factors may impact the separation performance via changing the condition number only. In this part, we attempt to analyze their relationships with the condition number in the analytical form and aim at reaching instructive conclusions. Moreover, special attention is paid to the case of singular mixtures.

Without the loss of generality, suppose that in Equation (13) d = *βλ*_1_, *λ*_1_ ≤ *λ*_2_ and *λ*_2_ = *Dλ*_1_. The parameter *β* represents the element spacing in a relative sense, *D* describes the divergence of wavelengths of the sources and the influence of the locations of sources will be depicted by the DOAs. With notations above, Equation (13) can be rearranged as:

(22) cond(A)=42−2cos[2π(βD)·(cosθ2cosφ2−Dcosθ1cosφ1)]

#### 3.2.1. Element Spacing of the Array

From Equation (22), it is obvious that for fixed *D* and DOAs, the condition number varies with *β* in a periodical way, with the period being:

(23) $$∆\beta =\left|\frac{D}{cos\theta_{2}cos\varphi_{2}-Dcos\theta_{1}cos\varphi_{1}}\right|,\;cos\theta_{2}cos\varphi_{2}-Dcos\theta_{1}cos\varphi_{1}\neq 0$$

The periodicity indicates that, increasing the element spacing of the array may have different impacts under varied situations depicted by *D* and DOAs.

Taking the partial derivative of Equation (23) against *D*, we have:

(24) $$\begin{array}{l} \frac{\partial ∆\beta }{\partial D}=\frac{\mathrm{sign}\left(cos\theta_{2}cos\varphi_{2}-Dcos\theta_{1}cos\varphi_{1}\right)\cdot cos\theta_{2}cos\varphi_{2}}{{\left(cos\theta_{2}cos\varphi_{2}-Dcos\theta_{1}cos\varphi_{1}\right)}^{2}},\\ \;\;\;\;\;cos\theta_{2}cos\varphi_{2}-Dcos\theta_{1}cos\varphi_{1}\neq 0 \end{array}$$

where sign(·) is the sign function. Hence, if *cosθ*_2_*cosϕ*_2_ > *cosθ*_1_*cosϕ*_1_, then the period increases along with the value of *D* within the interval $D\in [1,\frac{cos\theta_{2}cos\varphi_{2}}{cos\theta_{1}cos\varphi_{1}}),\;cos\theta_{1}cos\varphi_{1}\neq 0$; and decreases along with it once *D* is greater than$\frac{cos\theta_{2}cos\varphi_{2}}{cos\theta_{1}cos\varphi_{1}}$. If *cosθ*_2_*cosϕ*_2_ < *cosθ*_1_*cosϕ*_1_, the period decreases along with the value of *D*.

**Figure 3:**
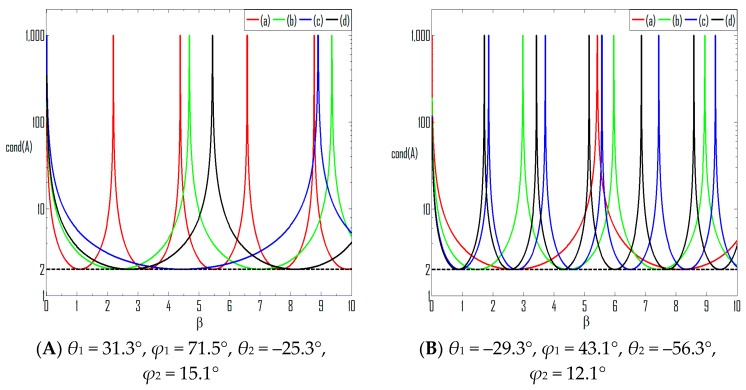
Condition number *vs.* element spacing. (**a**) *D* = 1.2; (**b**) *D* = 1.8; (**c**) *D* = 5.5; (**d**) *D* = 10.

The influence of the element spacing on the condition number is depicted by Figure 3.

The periodicity has been clearly demonstrated, with the period determined by varied *D* and DOAs. In Figure 3A, *θ*_1_ = 31.3°, *ϕ*_1_ = 71.5°, *θ*_2_ = −25.3°, *ϕ*_2_ = 15.1°, thus $\frac{cos\theta_{2}cos\varphi_{2}}{cos\theta_{1}cos\varphi_{1}}=3.2194$. We can see that for (a) and (b), where $D\in [1,\frac{cos\theta_{2}cos\varphi_{2}}{cos\theta_{1}cos\varphi_{1}})$, the period is lengthened with greater *D* ; while for (c) and (d), where $D>\frac{cos\theta_{2}cos\varphi_{2}}{cos\theta_{1}cos\varphi_{1}}$, the period is shortened with increasing *D*. In Figure 3B, *θ*_1_ = −29.3°, *ϕ*_1_ = 43.1°, *θ*_2_ = −56.3°, *ϕ*_2_ = 12.1°, and $\frac{cos\theta_{2}cos\varphi_{2}}{cos\theta_{1}cos\varphi_{1}}=0.8520$. We can see that the value of ∆β keeps decreasing with greater element spacing.

The minimal and maximal values of the condition number also arise periodically, and they appear respectively at *β*_min_ and *β*_max_ that equal:

(25) $$\begin{array}{l} \beta_{\min}=\frac{\left(m+\frac{1}{2}\right)D}{cos\theta_{2}cos\varphi_{2}-Dcos\theta_{1}cos\varphi_{1}}\\ \beta_{\max}=\frac{mD}{cos\theta_{2}cos\varphi_{2}-Dcos\theta_{1}cos\varphi_{1}} \end{array},\;cos\theta_{2}cos\varphi_{2}-Dcos\theta_{1}cos\varphi_{1}\neq 0,m\in \mathbb{Z}$$

According to previous analysis, the maximal values of the condition number which correspond to the singular mixtures should be avoided in applications, in order to prevent the critical failure of separation. In practice, the expected circumstance is that: with well-designed sensor arrays, the separation via ICA algorithms is satisfactory and can handle with mixtures consisted of sources with diverse carriers (within the band that the array is designed for) and from all possible locations. This is meaningful considering the intricate and unforeseen situations in applications. And here, we attempt to achieve this via designing properly the element spacing of the array.

One straightforward idea is that if the actual *β* is smaller than all possible *β*_max_, then the singular case would not occur. While *D* and DOAs are fixed, this indicates that:

(26) $$\begin{array}{l} \beta <\left|\frac{D}{cos\theta_{2}cos\varphi_{2}-Dcos\theta_{1}cos\varphi_{1}}\right|,\\ cos\theta_{2}cos\varphi_{2}-Dcos\theta_{1}cos\varphi_{1}\neq 0 \end{array}$$

Extending the relationship for all *D* ≥ 1 and *θ_i_* ∈[−90°, 90°]; $\varphi_{i}\in [-90^{o},\;90^{o}]$ yields:

(27) β<1

Since |*cosθ*_2_*cosϕ*_2_–*Dcosθ*_1_*cosϕ*_1_| ∈ (0,*D*).

This indicates that so long as the element spacing of the array is smaller than wavelengths of the sources, the cases of singular mixtures can be well prevented for sources with diverse carriers and from all possible directions. The only exception happens when cos*θ*_2_cos*ϕ*_2_ − *D*cos*θ*_1_cos*ϕ*_1_ = 0, then the mixture would be singular regardless of *β*. The conclusion can act as a meaningful guidance while designing the sensor array for applications adopting blind source separation. More specifically, if the array is designed for the frequency band (*f*_L_~*f*_H_), this means that the element spacing should satisfy *d* < *c*/*f*_H_. For instance, consider the satellite communication on the C band (4~8 GHz), then the element spacing should be designed below 37.5 mm for the sake of avoiding failures of separation. Moreover, if in the specific application, the DOAs of the sources can be further confined, say *θ*_1_*ϕ*_1_ ∈ [–*Ang*°, *An*g°] (*Ang* ≤ 90), then Equation (27) can be relaxed to:

(28) $$\beta <\frac{1}{1-\frac{f_{L}}{f_{H}}\cdot cos^{2}(Ang^{o})}$$

Notice that in many of previous works concerning independent component analysis via multi-sensor arrays, the element spacing is usually presumed as half of the wavelength of the sources, in both theoretical analysis and simulations (for instance, in [16,35]). However, the presumption may be invalid for actual scenarios. Because first, the accurate prior information of the wavelength cannot be obtained in most applications requiring ICA; second, since there may be radiators working on different carriers, the equality is impossible to be maintained. Hence, conclusions here provide a more practical instruction for the selection of the element spacing, and the traditional settlement can be regarded as one of its special case with *β* = 0.5.

#### 3.2.2. Carrier Frequencies of the NB Sources

From Equation (22), it can be deduced that the condition number varies with the reciprocal of *D* in a periodical way. The period is determined by *β* and DOAs as:

(29) $$∆\left(\frac{1}{D}\right)=\left(\frac{1}{\beta cos\theta_{2}cos\varphi_{2}}+\frac{cos\theta_{1}cos\varphi_{1}}{cos\theta_{2}cos\varphi_{2}}\right),\;cos\theta_{2}cos\varphi_{2}\neq 0$$

Take the partial derivative of Equation (29) against *β*, we have:

(30) ∂∆(1D)∂β=−1cosθ2cosφ2·1β2, cosθ2cosφ2≠0

Thus, the period decreases along with the increase of *β*.

Values of (1/*D*) correspond to minima (denoted as ((1/*D*)_min_)) and maxima (denoted as (1/*D*)_max_))) of the condition number that can be obtained as:

(31) (1D)min=(m+12)+βcosθ1cosφ1βcosθ2cosφ2(1D)max=m+βcosθ1cosφ1βcosθ2cosφ2, m∈ℤ, cosθ2cosφ2≠0

**Figure 4:**
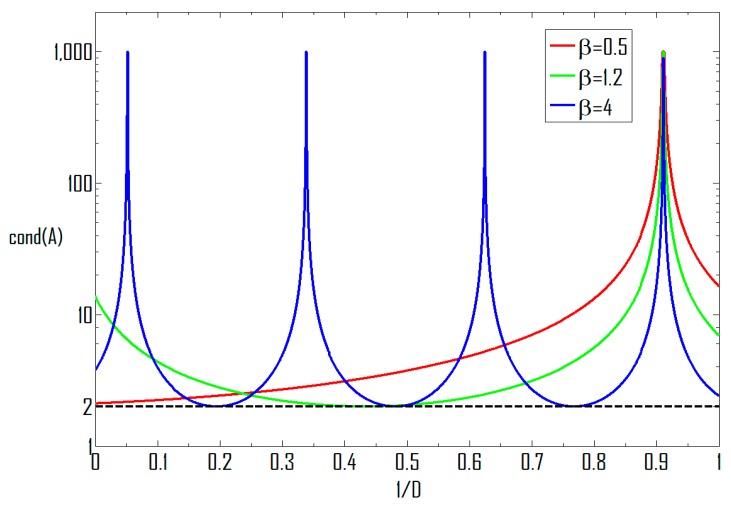
Condition number *vs.* the divergence of carrier frequencies.

Since (1/*D*) ∈ (0, 1], the number of possible minimum and maximal points are determined by *β* and DOAs as well. The way the condition number varies with (1/*D*) is demonstrated in Figure 4. It can be seen that the value of ∆(1/*D*) decreases with greater *β* and since *θ*_1_ = 31.3°, *ϕ*_1_ = 21.5°, *θ*_2_ = −25.3°, *ϕ*_2_ = 15.1°, here, we can see that for *β* = 4, the possible (1/*D*)_min_) and (1/*D*)_max_) are 0.1948, 0.4812, 0.7676 and 0.0516, 0.3380, 0.6244, 0.9108, respectively, and since *cosθ*_2_*cosϕ*_2_ > *cosθ*_1_*cosϕ*_1_, a maximal point appears at *D* = (*cosθ*_2_*cosϕ*_2_/*cosθ*_1_*cosϕ*_1_) regardless of *β*. This is consistent with the conclusion obtained in Section 3.2.1.

Though Equation (31) indicates values of (1/*D*) correspond to the maxima of the condition number, it should be noticed that a maximal point is valid only when it is within the interval of (0, 1]. This means that *m* which relates to a valid (1/*D*)_max_ should satisfy:

(32) $$\begin{array}{c} -\beta cos\theta_{1}cos\varphi_{1}<m\leq \beta cos\theta_{2}cos\varphi_{2}-\beta cos\theta_{1}cos\varphi_{1},\\ m\in \mathbb{Z},\;cos\theta_{2}cos\varphi_{2}\neq 0 \end{array}$$

If *β* > 1, it should be obvious that there must exist *m*∈[⌈–*βcosθ*_1_*cosϕ*_1_⌉ + 1, ⌊*βcosθ*_2_*cosϕ*_2_ – *βcosθ*_1_*cosϕ*_1_⌋] for certain groups of (*θ*_i_,*ϕ_i_*) (*i* = 1,2), that would validate Equation (32). If *β* = 1, then the valid *m* would be 1 for *cosθ*_1_*cosϕ*_1_ = 0, or 0 for *cosθ*_1_*cosϕ*_1_≠ 0. If 0 < *β* < 1, then for *cosθ*_1_*cosϕ*_1_ = 0, there would not be *m* ∈ Z satisfying Equation (32); for *cosθ*_1_*cosϕ*_1_ ≠ 0, the only valid *m* would be 0, with (1/*D*)_max_ = *cosθ*_1_*cosϕ*_1_/*cosθ*_2_*cosϕ*_2_. Notice that this is consistent with the analysis on the element spacing, where it has been stated that if *β* < 1, the cases of singular mixtures would not occur for all *D* ≥ 1 and *θ_i_* ∈ [–90°, 90°], *ϕ_i_*∈[–90°, 90°], with the only exception of *cosθ*_2_*cosϕ*_2_ − *Dcosθ*_1_*cosϕ*_1_.

From Equation (31), it could also be found that the minimum of (1/*D*)_max_ (denoted as min(1/*D*)_max_) is:

(33) $$\left\{ \begin{array}{l} \frac{-\left\lfloor \beta cos\theta_{1}cos\varphi_{1}\right\rfloor +\beta cos\theta_{1}cos\varphi_{1}}{\beta cos\theta_{2}cos\varphi_{2}},\;\beta cos\theta_{1}cos\varphi_{1}\notin \mathbb{Z}\\ \frac{1}{\beta cos\theta_{2}cos\varphi_{2}},\;\beta cos\theta_{1}cos\varphi_{1}\in \mathbb{Z} \end{array}\right.$$

where ‘[x]’ indicates the largest integer no greater than *x*.

This indicates that for certain *β* and (*θ_i,_ϕ_i_*) (*i* = 1,2), the cases of singular mixtures could be avoided by sufficiently large values of *D*, the reciprocal of which is smaller than min(1/*D*)_max_. Further, for the adaptation of varied (*θ*_2,_*ϕ*_2_), Equation (33) could be transferred to:

(34) $$\left\{ \begin{array}{l} \frac{-\left\lfloor \beta cos\theta_{1}cos\varphi_{1}\right\rfloor +\beta cos\theta_{1}cos\varphi_{1}}{\beta },\;\beta cos\theta_{1}cos\varphi_{1}\notin \mathbb{Z}\\ \frac{1}{\beta },\;\beta cos\theta_{1}cos\varphi_{1}\in \mathbb{Z} \end{array}\right.$$

Equations (33) and (34) could be meaningful in the situation where one of the sources is fixed in both of the frequency and its location, while the other source is a moving one. Then it can be told from the two formulas how separated the other source should be from the fixed one in terms of carrier frequencies, to ensure the success of their separation while it is moving within certain region. Such scenario may exist in the acoustic applications with one of the speaker walking around the microphone.

#### 3.2.3. Locations of the Sources

First, it has been revealed by Equation (22) one of the edges of separation via ICA algorithms over spatial filtering (beamforming), that is: When sources arrive from identical (or very close) directions, the separation based on spatial filtering will become invalid; while it is still possible for ICA algorithms to work, so long as there is difference in carriers of the NB sources (*D* ≠ 1).

Second, it can be seen that sources from varied locations may correspond to identical condition numbers, so long as their DOAs result in the same value of *cosθ_i_cosϕ_i_* (*i* = 1,2). More specifically, for fixed *β*, *D* and (*θ*_1_,*ϕ*_1_), the mixing matrix would be singular so long as:

(35) $$cos\theta_{2}cos\varphi_{2}=\frac{mD}{\beta }+Dcos\theta_{1}cos\varphi_{1},\;m\in \mathbb{Z}$$

**Figure 5:**
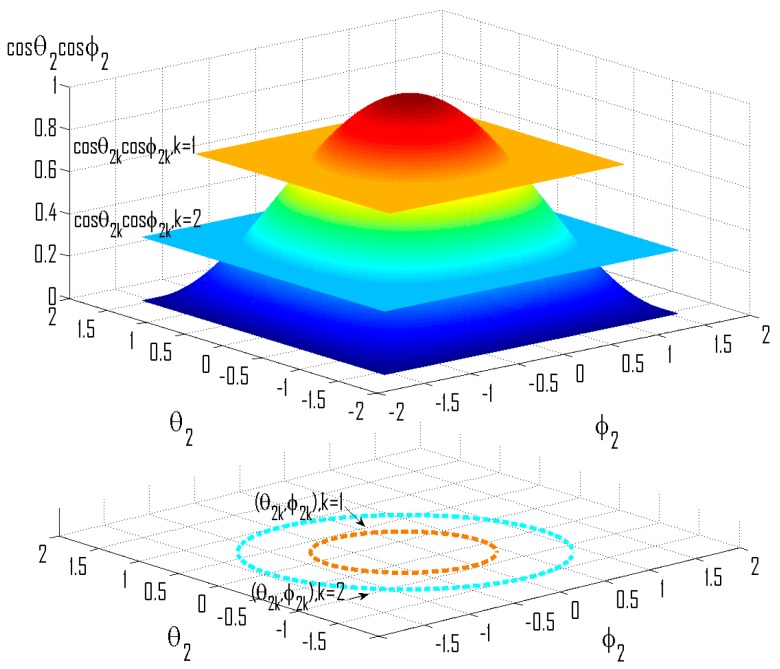
Distribution of locations of the source correspond to singular mixtures.

Since *cosθ*_2_*cosϕ*_2_ ∈ [0, 1], the value of possible *m* should be within [−*βcosθ*_1_*cosϕ*_1_, (*β*/*D*) – *βcosθ*_1_*cosϕ*_1_]. Suppose that the set of valid *m* is {*m_k_*, *k* = 1,2,…}, then for each of its element there are correspondingly a group of (*θ*_2*k*_,*ϕ*_2*k*_) satisfying *cosθ*_2*k*_*cosϕ*_2*k*_ = (*Dcosθ*_1_*cosϕ*_1_ + *m_k_*∙*D*/*β*), and this is further depicted by Figure 5.

To avoid the situation where sources from with certain groups of DOAs become inseparable, controls are necessary on the interval [−*βcosθ*_1_*cosϕ*_1_, (*β*/*D*) – *βcosθ*_1_*cosϕ*_1_], to reduce the number of integers it includes.

In terms of the element spacing, if *β* < 1, then –*βcosθ*_1_*cosϕ*_1_ ∈(−1, 0] and (*β*/*D*) − *βcosθ*_1_*cosϕ*_1_ ∈ (−1,1), thus the only possible *m_k_* = 0 and the corresponding *cosθ*_2*k*_*cosϕ*_2*k*_ = *Dcosθ*_1_*cosϕ*_1_, while *Dcosθ*_1_*cosϕ*_1_ ∈ [0, 1]. On the other hand *D* > *β* for *βcosθ*_1_*cosϕ*_1_ ∈ Z, then the valid *m_k_* = −*βcosθ*_1_*cosϕ*_1_ and the mixture may turn singular for *cosθ*_2_*cosϕ*_2_ = 0 only; if $D>\frac{\beta }{\beta cos\theta_{1}cos\varphi_{1}-\left\lfloor \beta cos\theta_{1}cos\varphi_{1}\right\rfloor }$ for *βcosθ*_1_*cosϕ*_1_ ∉ Z, then since (*β*/*D*) − *βcosθ*_1_*cosϕ*_1_ < ⌊*βcosθ*_1_*cosϕ*_1_⌋, the valid *m_k_* does not exist. It can be seen that the analysis here is consistent with the conclusions presented in Section 3.2.1 and Section 3.2.2.

For situations where values of *β* and *D* can be roughly determined beforehand, Equation (35) might provide helpful guidance in the arrangement of the sensors (such as the microphone) in terms of directivities and locations, to possibly avoid singular mixtures.

**Figure 6:**
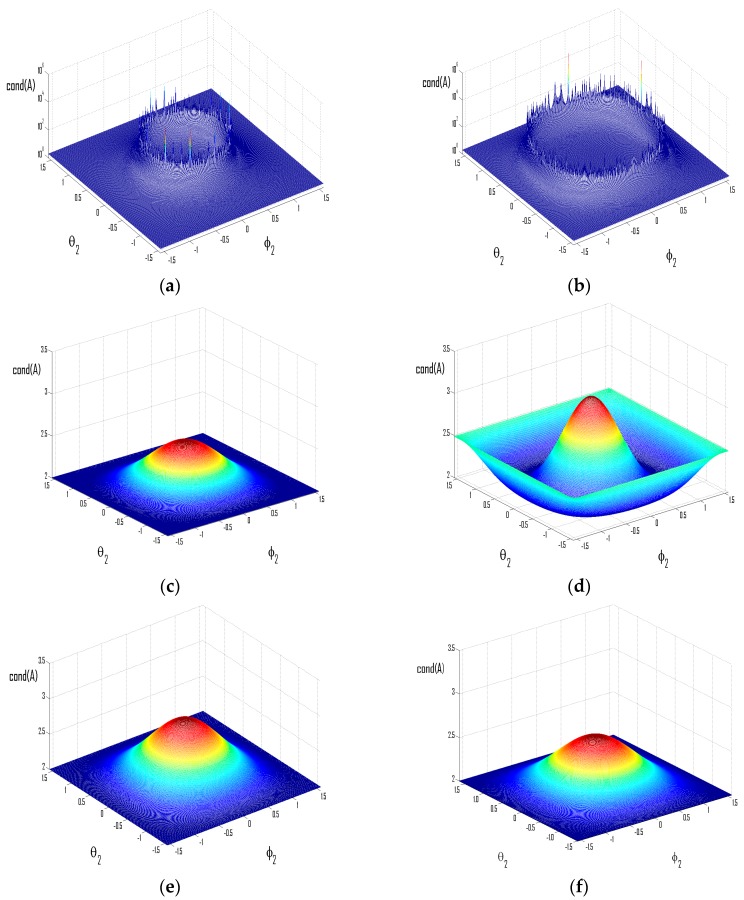

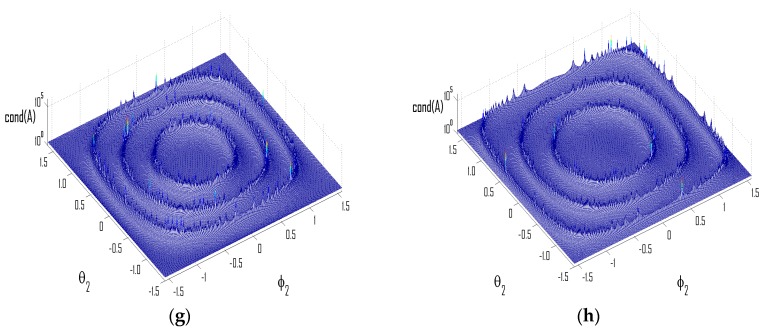
Condition number for sources from varied locations. (**a**) *β* = 2, *D* = 3, (*θ*_1_,*ϕ*_1_) = (30°,30°); (**b**) *β* = 2, *D* = 2, (*θ*_1_,*ϕ*_1_) = (–40°,–15°); (**c**) *β* = 0.7, *D* = 3, (*θ*_1_,*ϕ*_1_) = (30°,30°); (**d**) *β* = 0.95, *D* = 2, (*θ*_1_,*ϕ*_1_) = (–40°,–15°); (**e**) *β* = 2, *D* = 8, (*θ*_1_,*ϕ*_1_) = (30°,30°); (**f**) *β* = 2, *D* = 10, (*θ*_1_,*ϕ*_1_) = (–40°,–15°); (**g**) *β* = 5, *D* = 1.5, (*θ*_1_,*ϕ*_1_) = (30°,30°); (**h**) *β* = 3, *D* = 1.009, (*θ*_1_,*ϕ*_1_) = (–40°,–15°).

In Figure 6, condition numbers under varied DOAs have been shown while representative values of *β* and *D* are set for the sake of illustrating conclusions obtained in this section. In Figure 6a, the condition number becomes extremely large for a group of (*θ*_2_,*ϕ*_2_) distributed in a circle centered on the origin. Since [–*βcosθ*_1_*cosϕ*_1_, (*β*/*D*) − *βcosθ*_1_*cosϕ*_1_] = [−1.5, −0.83] therein and valid *m* = −1. It is similar for Figure 6b where [–*βcosθ*_1_*cosϕ*_1_, (*β*/*D*) − *βcosθ*_1_*cosϕ*_1_] = [−1.48, −0.48]. Notice that in Figure 6g,h, there exist more circles consisting of singular points, since the set of valid *m* are {−3,−2,−1} and {−2,−1,0}, respectively. In Figure 6c,d, *β* is set to be smaller than 1, and *Dcosθ*_1_*cosϕ*_1_ > 1, thus it can be seen that singular cases have been effectively avoided. In e and f, the value of *D* is increased remarkably, and hence, original singular cases are successfully removed. For example, in Figure 6e *βcosθ*_1_*cosϕ*_1_ ∉ Z and *D* > (*β*/*βcosθ*_1_*cosϕ*_1_, −⌊ *βcosθ*_1_*cosϕ*_1_⌋) = 4, thus according to the previous analysis, there would not be singular points. To conclude, it has been demonstrated in Figure 6 the validity of conclusions obtained in this section.

### 3.3. Countermeasure for Singular Mixtures

For most applications, the occurrence of (nearly) singular mixtures would be disastrous, and the intricate actual scenarios have made it somehow inevitable. And here, we attempt to propose certain countermeasure against such situations to avoid extremely poor separation performance.

According to Equation (20), reducing the power of noise could be helpful, possibly via means of band pass filtering. Hence, more effective actions may be taken in terms of changing the condition number.

One straightforward idea is that: when the mixing process is (nearly) singular, certain extra phase delay *Ψ* could be introduced into the observations. This could be effective since when the mixtures are (nearly) singular, 2*π*(*β*/*D*)·(*cosθ*_2_*cosϕ*_2_ – *Dcosθ*_1_*cosϕ*_1_) equals or is quite close to 2*mπ*, *m*∈Z, and the value of the condition number after the introduction of the extra phase delay would be:

(36) cond(A)=42−2cos[2π(βD)·(cosθ2cosφ2−Dcosθ1cosφ1)+ψ]≈42−2cosψ

Thus, by setting the proper value of *Ψ* the revised condition number would be more beneficial for the separation, and the ideal value of *Ψ* would be (2*m* + 1)*π*, *m*∈Z. Notice that this could not be realized via directly shifting the phase of the mixtures, since it can be deduced from Equations (8) and (10) that this actually would not change the condition number. In practice, *Ψ* could be introduced through shifting the mixtures along the axis of the observation time.

**Figure 7:**
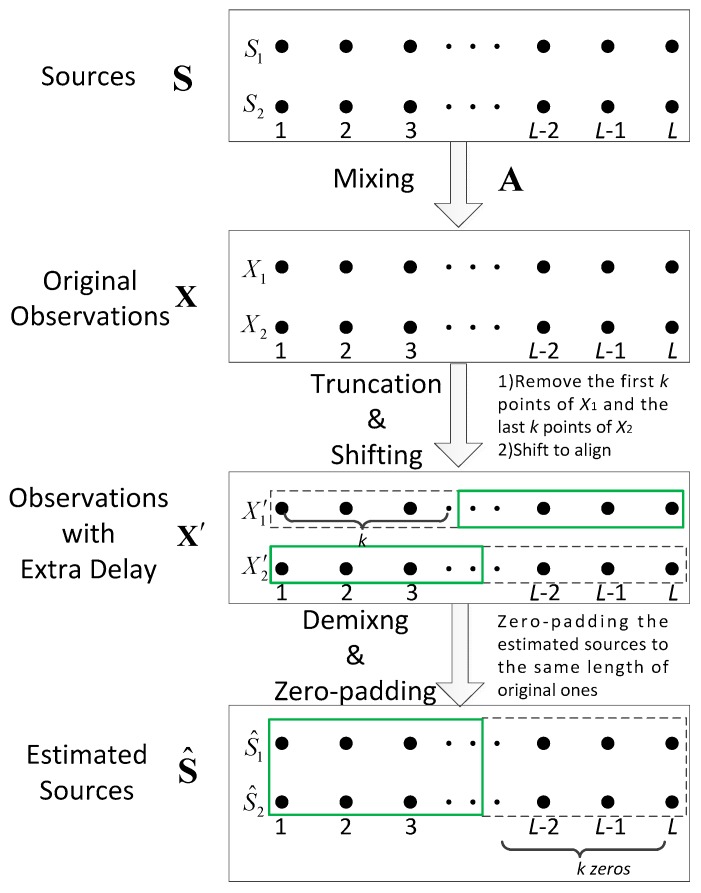
The proposed scheme against singular mixtures.

More specifically, consider the following scheme depicted by Figure 7. The idea here is that the introduction of extra phase delay in Equation (22) is achieved with one of the observations undergoing an artificial time-lag. For instance, if the observation from the second sensor is shifted backward by *k* points, then the equivalent phase delay introduced is:

(37) $$\psi =2\pi kT_{s}(f_{2}-f_{1})$$

in which *T_s_* is the sample interval.

It should be stated that the proposed scheme is only designed to combat the situations of (nearly) singular mixtures. In fact, only when originally 2*π*(*β*/*D*)·(*cosθ*_2_*cosϕ*_2_–*Dcosθ*_1_*cosϕ*_1_) is close to 2*mπ*, *m*∈Z can it be ensured that the extra phase delay is constructive rather than destructive. One of the actual problems is judging the singularity of the current observations and deciding whether the proposed procedure is needed. Since the condition number of the mixing matrix and the SINR at the output is generally hard to obtain, the correlation coefficient *ρ* can act as one practical criterion [34]. For random variables *x*(*ξ*) and *y*(*ξ*), their correlation coefficient is defined as:

(38) $$\rho_{xy}=\frac{c_{xy}}{\sigma_{x}\sigma_{y}}$$

in which *c_xy_* is the cross covariance of *x*(*ξ*) and *y*(*ξ*), $\sigma_{x}^{2}$ and $\sigma_{y}^{2}$ are the variances of them.

The idea is that when the original mixing matrix is (nearly) singular, it can be seen from Equation (8) that the two observations would be (approximately) coherent, and the module value of the correlation coefficient between the two observations would approach 1. More specifically, since the sources are presumed to be zero-mean and independent, it can be calculated from Equations (8) and (38) that the module value of the correlation coefficient between the two observations (denoted as *ρ_x_*_1*x*2_) equals:

(39) $$\left|\rho_{x_{1}x_{2}}\right|=\frac{1}{\sigma_{s_{1}}^{2}+\sigma_{s_{2}}^{2}}\sqrt{\sigma_{s_{1}}^{4}+\sigma_{s_{2}}^{4}+2\sigma_{s_{1}}^{2}\sigma_{s_{2}}^{2}\cdot \left(1-\frac{8}{cond{\left(A\right)}^{2}}\right)}$$

in which $\sigma_{s_{1}}^{2}$ and $\sigma_{s_{2}}^{2}$ are the variances of the sources. Hence, it can be seen that while *cond*(**A**) = ∞, $\left|\rho_{x_{1}x_{2}}\right|=1$; and while *cond*(**A**) = 2, $\left|\rho_{x_{1}x_{2}}\right|=\frac{\sigma_{s_{1}}^{2}-\sigma_{s_{2}}^{2}}{\sigma_{s_{1}}^{2}+\sigma_{s_{2}}^{2}}$; if $\sigma_{s_{1}}^{2}=\sigma_{s_{2}}^{2}$, then for *cond*(**A**) = 2, $\left|\rho_{x_{1}x_{2}}\right|=0$, which means then the two observations are orthogonal.

In practice, a threshold associated with the noise level and the number of samples can be set based on experience, and when the module value of the correlation coefficient between the observations exceeds the threshold, the mixtures can be regarded as (nearly) singular. And then the proposed scheme should be adopted. During our simulations, it is found that generally speaking k=1 or 2 should be suitable under most scenarios.

## 4. Simulations

In this section, experiments are carried out using Intel(R) Core (TM) 2 Duo CPU E6550 @ 2.33 GHz and MATLAB 7.12.0(R2011a).

Experiment 1: Separation performance of ICA algorithms under varied noise levels and the singularity of the mixing matrix.

In this experiment, quadrature phase shift keying (QPSK) and binary phase shift keying (BPSK) signals are adopted as sources with carrier frequencies 0.38, 0.127 MHZ, and symbol rates 2, 1 Kbps, respectively. The sample rate is set as 1 MHz, and the observation length is 1024. The receiving array consists of two sensors with the element spacing being two times the wavelength of source 1. The DOA of source 1 is (25.1°, 40.5°), while the elevation angle of source 2 is 27.9°.

The SNR at the input of the separator and the SINRM adopted here as the measurement for separation performance are defined as in [35]. Typical ICA algorithms tested include: FastICA [22], EASI [23] and Infomax [24]. The initialization of the demixing matrix is generated randomly. The non-linear function selected for FastICA is *G*(*y*) = log(0.1 + *y*), with more options listed in [22]; the adaptation step for EASI is 0.01, and the nonlinearity is chosen as *g_i_*(**y**) = |*y_i_*|^2^*y_i_* for 1 ≤ *i* ≤ *P*; the updating step for Infomax is 0.1, and the nonlinearity is selected in the form of *g_i_*(*y*) = –|*y_i_*|^*α_i_* – 1^sign(*y*), 1 ≤ *i* ≤ *P*, with *α_i_ = 2*. Results are obtained with 500 times of Monte Carlo simulations.

**Figure 8:**
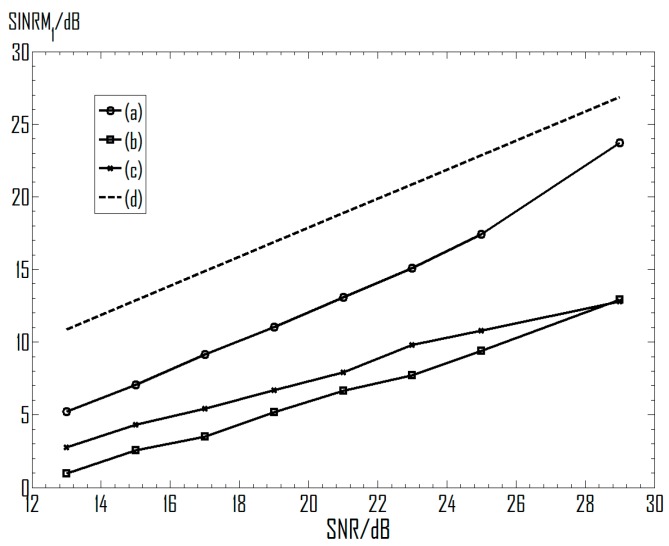
Separation performance under varied input SNRs. (**a**) FastICA; (**b**) EASI; (**c**) Informax; (**d**) Equation (20).

Figure 8 shows curves of SINRM_1_ varying with the input SNR, in which the azimuth angles of source 2 is −81.1°, and thus the condition number is 2.56. It can be seen that for these representative ICA algorithms, their performances measured by the SINR increase along with input SNR. More specifically, curves of the three algorithms are roughly parallel with the theoretical upper limit indicated by (d), which means that their output SINRs vary almost linearly with the input SNR. Take the FastICA as an example, while the SNR increases from 19 dB to 21 dB, the output SINR increases from 11.04 dB to 13.06 dB.

**Figure 9:**
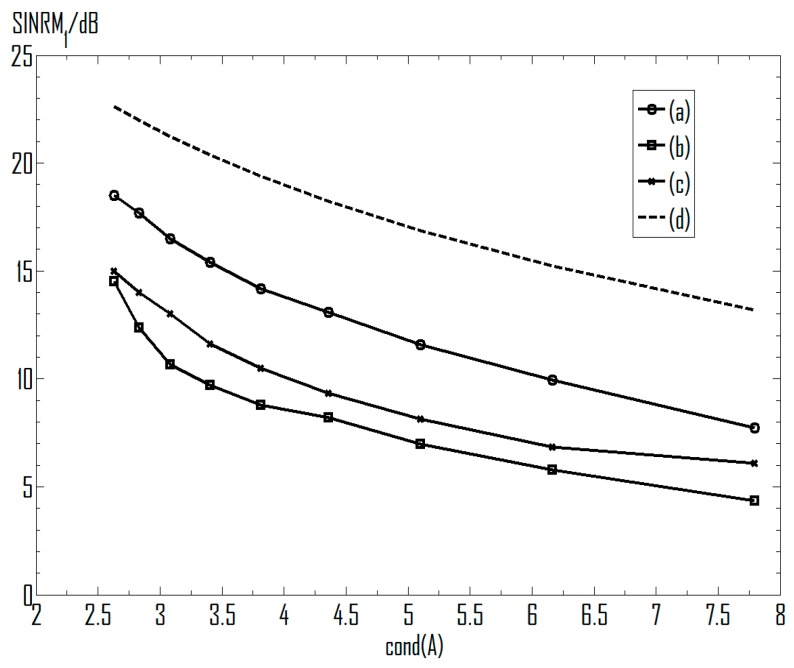
Influence of the condition number on the separation performance. (**a**) FastICA; (**b**) EASI; (**c**) Informax; (**d**) Equation (20).

Figure 9 demonstrates the SINRM_1_ under varied condition numbers, and the different condition numbers are controlled by the azimuth angle of source 2. It can be seen that the SINRs measuring the performance decrease along with the increase of the condition number. Curves of their performance are all below the theoretical line based on Equation (20), and are varying in a nearly parallel way with the curve (d). For instance, in curve (a), the output SINR decreases by 2.21 dB (from 9.93 dB to 7.72 dB) when the condition number increases from 6.17 to 7.79. This is close to the result obtained with Equation (20), since 20log(7.79/6.17) ≈ 2.03.

In general, results obtained in this experiment have demonstrated well the influence of the environmental noise and the condition number on the separation performance of typical ICA algorithms, and do not conflict with the theoretical limitation indicated by Equation (20).

Experiment 2: Separation performance and practical factors.

**Figure 10:**
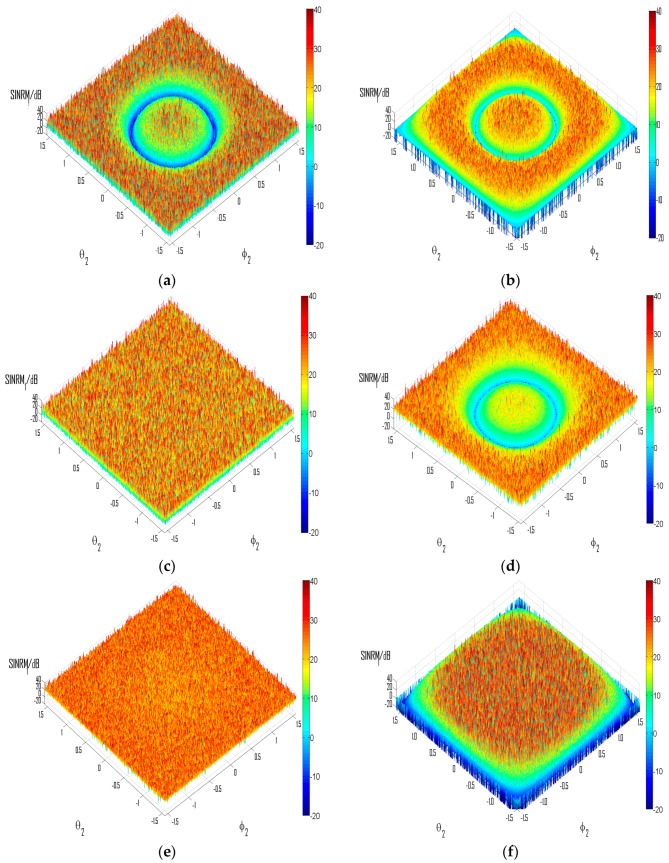
Separation performance of typical ICA algorithm under varied scenarios. (**a**) *β* = 2, *D* = 3; (**b**) *β* = 2, *D* = 1.5; (**c**) *β* = 0.7, *D* = 3; (**d**) *β* = 0.85, *D* = 1.5; (**e**) *β* = 2, *D* = 8; (**f**) *β* = 2, *D* = 3.

In this experiment, the same sources have been adopted, except that the carrier frequency of source 2 is set according to different values of *D* tested. The DOA of source 1 is (30°, 30°) in Figure 10a,c,e, and (−45°, 45°) in Figure 10b,d,f. The performances have been demonstrated with representative values of *β* and *D*, as well as for source 2 from all possible directions. The input SNR is set at 30 dB. Since it has been demonstrated in Figure 8 and Figure 9 that performances of typical ICA algorithms are all influenced by the condition number in ways alike, only results from the FastICA algorithm as described in Experiment 1 are presented, while similar results have been obtained with other ICA algorithms mentioned in Experiment 1 during our simulations.

Figure 10 demonstrates the SINRM_1_ under varied practical factors simulating possible actual scenarios. It can be seen that in Figure 10a,b, the separation performance endures dramatic deterioration (at least 15 dB) for source 2 from certain groups of directions. This is because in Figure 10a, [–*βcosθ*_1_*cosϕ*_1_, (*β*/*D*) – *βcosθ*_1_*cosϕ*_1_] = [–1.5, –0.83], thus *m* = –1 is valid as analyzed in Section 3.2.2, and the deterioration of performance occurs for *cosθ*_2_*cosϕ*_2_ = (*Dcosθ*_1_*cosϕ*_1_ – *D*/*β*); for Figure 10b, valid *m* = [–1, 0], and thus corresponding *cosθ*_2_*cosϕ*_2_ = [0, 0.75] (notice that groups of (*θ*_2_,*ϕ*_2_) correspond to *cosθ*_2_*cosϕ*_2_ = 0 distribute on the edge of Figure 10b). In Figure 10c,d, *β* is reduced to be smaller than 1. For Figure 10c, since *Dcosθ*_1_*cosϕ*_1_ > 1, the singular cases have been completely avoided for source 2 from all possible directions; while in Figure 10d, *Dcosθ*_1_*cosϕ*_1_ < 1, thus the mixture would turn singular for *cosθ*_2_*cosϕ*_2_ = *Dcosθ*_1_*cosϕ*_1_, though with *β* < 1.

In Figure 10e,f, the value of *D* is set according to Equation (34). In Figure 10e, since *βcosθ*_1_*cosϕ*_1_ ∉ Z, *D* is set to be greater than (*β*/*βcosθ*_1_*cosϕ*_1_,–⌊ *βcosθ*_1_*cosϕ*_1_⌋) = 4; in Figure 10f, *βcosθ*_1_*cosϕ*_1_ = 1, thus *D* > *β* > 2 should be enough for the prevention of singular cases and we set *D* = 3; and it should be noticed herein that the mixture would still become singular for *cosθ*_2_*cosϕ*_2_ = 0, which is straightforward from Equation (22). To conclude, the experiment has validated the conclusions obtained in Section 3.2 about the influence of fundamental practical factors on the separation performance, especially those concerned the cases of singular mixtures.

Experiment 3: Effectiveness of the proposed countermeasure against singular mixtures.

**Figure 11:**
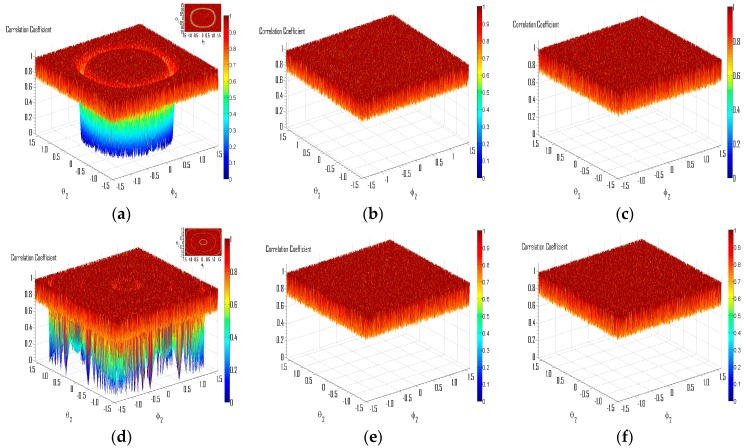
Effectiveness of the proposed countermeasures. (**a**) *β* = 0.75, without extra phase delay (**b**) *β* = 0.75, k = 1 (**c**) *β* = 0.75, k = 2 (**d**) *β* = 4.5, without extra phase delay (**e**) *β* = 4.5, k = 1 (**f**) *β* = 4.5, k = 2.

In this experiment, the carrier frequencies of the QPSK and BPSK sources are set at 0.4 MHz and 0.2 MHz respectively, thus *D* = 2. The DOA of source 1 is (56°, 63°). The sampling frequency is 1.2 MHz and the length of observation is 5120. The input SNR is 30 dB. After numerous experiments, the threshold of the correlation coefficient for judging the singular cases is chosen as 0.99. In Figure 11, the correlation coefficient between source 1 and its corresponding restitution component is adopted as other measurement of performance. In Figure 11a–c, *β* = 0.75; and in Figure 11d–f, *β* = 4.5. Figure 11a,d demonstrate the separation performance, without the proposed countermeasure. It can be seen that the restitution quality is unsatisfactory while source 2 is from certain directions (the distribution of such DOAs of source 2 can be seen clearer in the subplot northeast of Figure 11a,d). In Figure 11b,e, the proposed countermeasure has been adopted and *k* = 1. Thus, the extra phase delay introduced for these singular cases is *Ψ*_1_ = 2*πkT_s_*(*f*_2_ − *f*_1_) = *π*/3; and since *k* = 2 in Figure 11c,f, the extra delay therein is *Ψ*_2_ = 2*π*/3. It can be seen that restitution qualities in the originally singular points have been dramatically improved, and the failure of separation has been effectively avoided. Thus, the effectiveness of the proposed countermeasure for the singular mixtures has been validated.

**Figure 12:**
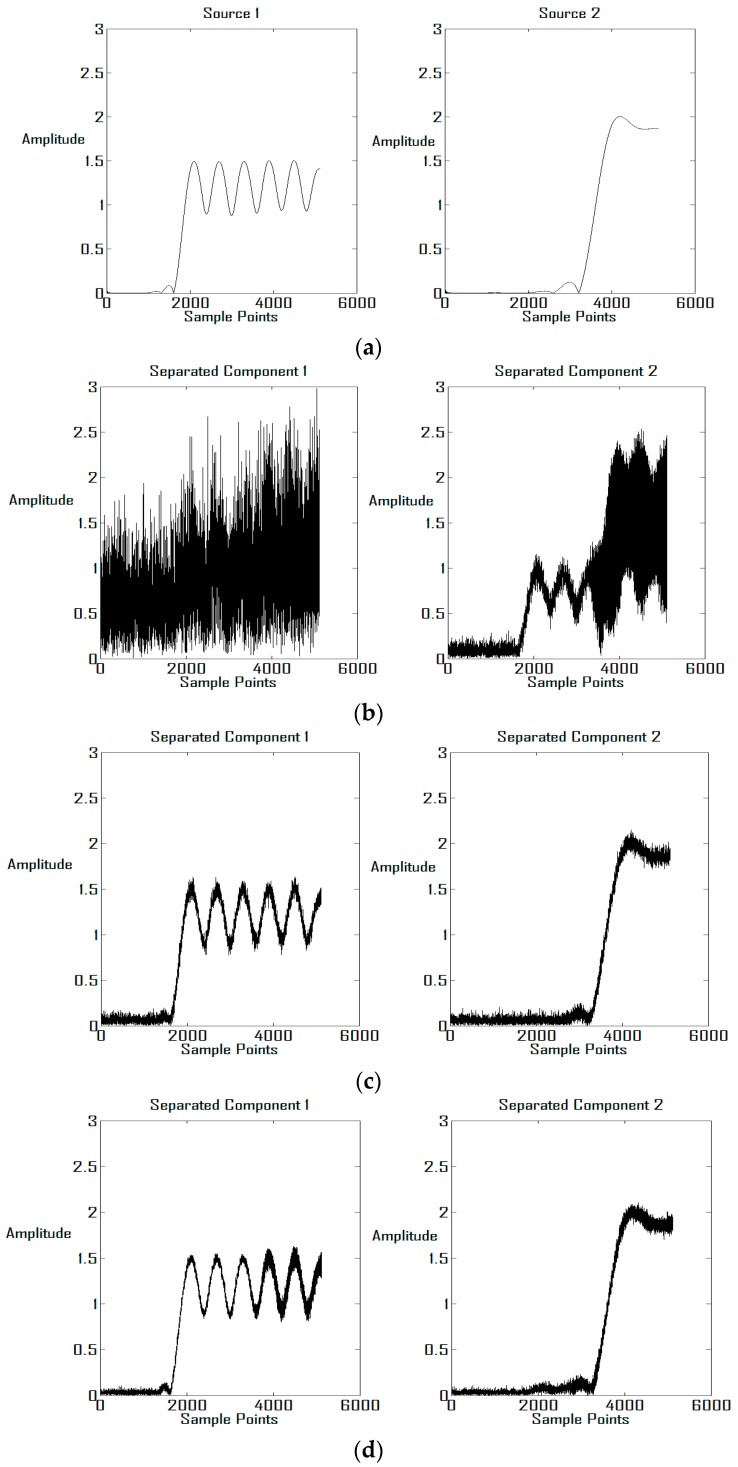

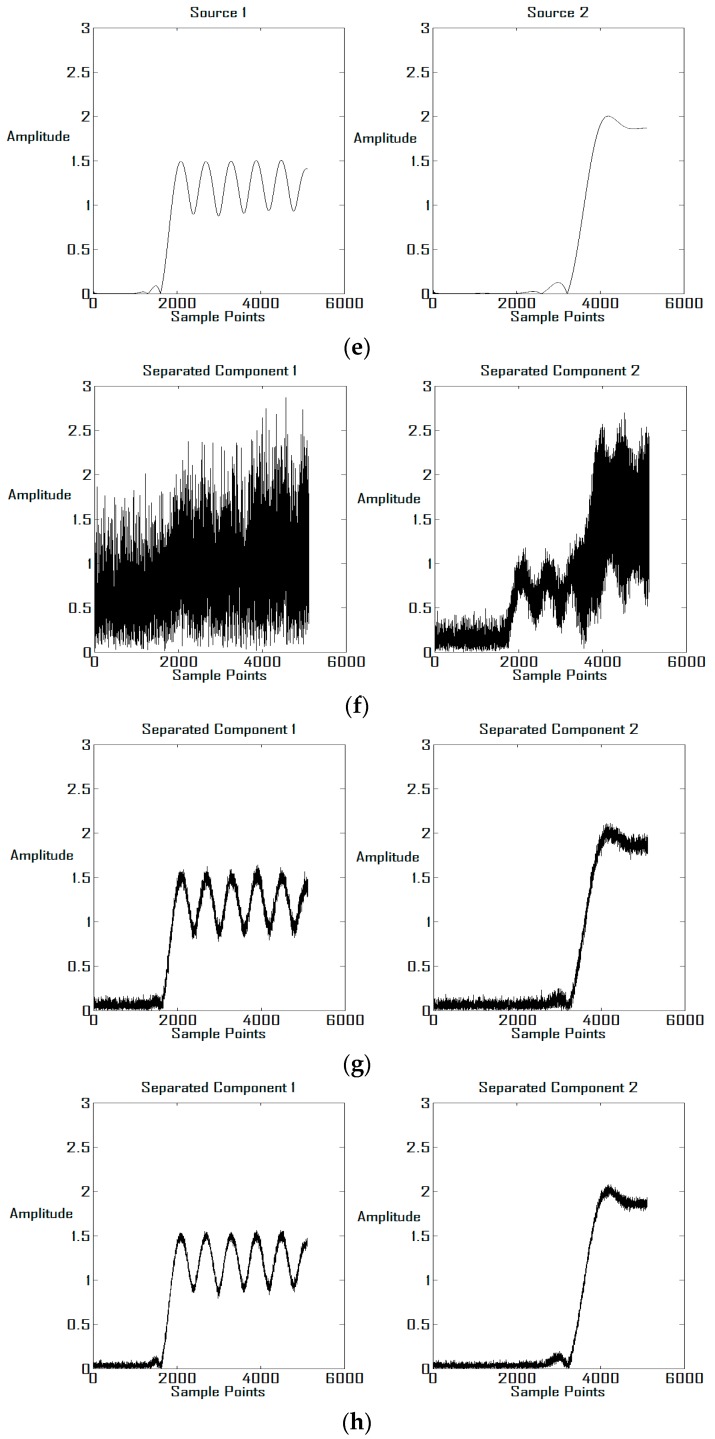
Restitution quality with the proposed scheme. (**a**) Original Sources (**b**) Recovered components, without extra phase delay (**c**) Recovered components, k = 1 (**d**) Recovered components, k = 2 (**e**) Original Sources (**f**) Recovered components, without extra phase delay (**g**) Recovered components, k = 1 (**h**) Recovered components, k = 2.

In Figure 12, the waves of the sources and their estimations are demonstrated to show the improvement of the restitution quality in a more explicit way. Figure 12a presents the original sources in Figure 11a–c. In Figure 12b, the recovered components for DOA of source 2 being (−11.5°, 61.2°) (the corresponding condition number is 46.7) have been presented, and we can see that the distortion in the wave forms is unbearable. In Figure 12c,d, the proposed scheme is applied for the same situation, and it is obvious that the restitution quality has been improved significantly. Similar analysis can be done for Figure 12e–h, which correspond to Figure 11d–f.

## 5. Conclusions

In this paper, the performance of ICA algorithms in sensor arrays is fully discussed. First, the analytic connection between the environment noise variance, the source variance, the condition number of the mixing matrix and the optimal signal to interference-plus-noise ratio is deduced in Equation (20). It indicates quantitatively how the separation performance would be determined by the environment noise level and the singularity of the mixing process. Next, the relationships between several fundamental practical factors in sensor array receiving and the condition number are analyzed in detail. Factors concerned include the element spacing of the array (depicted by the parameter *β*), the frequencies (depicted by the parameter *D*) and locations of the sources (depicted by the DOAs of sources). Generally speaking, it is found that: (1) the condition number varies with the element spacing in a periodical way, with the period is determined by *D* and DOAs; (2) the condition number also varies with the reciprocal of *D* periodically, and the period is determined by *β* and DOAs; (3) as for the locations of sources, we discover that sources from varied locations may lead to identical condition numbers, so long as their DOAs result in the same value of *cosθ_i_cosϕ_i_* (*i* = 1,2). Considering its significance for applications, the situations where the mixtures become singular have been paid special attention to. Main conclusions concerning the prevention of singular cases include: (1) the cases of singular mixtures can be well prevented for sources with diverse carriers and from all possible directions, so long as the element spacing of the array is smaller than wavelengths of the sources. And the only possible exception exists when *cosθ*_2_*cosϕ*_2_ − *Dcosθ*_1_*cosϕ*_1_ = 0, then the mixture would be singular regardless of *β*; (2) for certain *β* and (*θ_i_*,*ϕ_i_*)(*i* = 1,2), the cases of singular mixtures could be avoided by sufficiently large values of *D*, the reciprocal of which is smaller than min(1/*D*)_max_ in Equation (34). Moreover, a countermeasure for the singular cases has been proposed, based on the introduction of extra phase delay. And in reality, the scheme is realized with one of the observations undergoing artificial time-shifting. Conclusions and the effectiveness of the proposed countermeasure are validated by succeeding experiments. As a whole, conclusions and results obtained in this paper could be instructive while applying ICA algorithms in sensor arrays or designing the receiving array, especially for the prevention of critical failure in separation. Future works may focus on analyzing the effects of more complicated practical factors such as the multi-path effect or array calibration imperfection, which would involve more complex model of the array output and coupling among different kinds of factors. Also, the effectiveness of the proposed scheme in Section 3.3 could be further promoted, possibly based on the interpolation of the observations to allow better control on the value of the extra phase delay by changing the sample interval. Since the desired phase delay is (2*m* + 1)*π*, *m* ∈ Z in theory.
